# Artificial intelligence in orthopedic trauma surgery: a scoping review of current applications and research gaps

**DOI:** 10.1007/s00402-026-06276-6

**Published:** 2026-03-28

**Authors:** Lennard M. Wurm, Wolfgang Ertel, Dominik Laue

**Affiliations:** 1 Department of Traumatology and Reconstructive Surgery, Charité - University Medicine Berlin, Berlin, Germany; 2 Medical Faculty, Heinrich Heine University Düsseldorf, Düsseldorf, Germany

## Abstract

**Background:**

Artificial intelligence (AI) is rapidly transforming clinical decision-making, yet its role in orthopedic trauma surgery remains fragmented and unevenly validated in clinical practise.

**Methods:**

We conducted a PRISMA-SCR–compliant scoping review using a systematic search of the Semantic Scholar, OpenAlex and PubMed corpus via Elicit (497 records). Studies were eligible if they applied AI or machine-learning methods to traumatic orthopedic conditions, included ≥ 10 human subjects, reported quantitative performance metrics, and represented original research. After title/abstract and full-text screening, 146 studies were included. Data on study characteristics, AI methodology, clinical application, validation strategy, performance metrics, explainability and translational maturity were synthesized descriptively.

**Results:**

Research output increased sharply after 2017, with 52% of all studies published since 2022. Most studies were retrospective (≈ 99%). Deep learning dominated the field (61%), particularly for fracture detection and classification, while classical machine-learning models were mainly used for outcome prediction. Internal validation was reported in 85% of studies, whereas only 15% clearly performed external or multicenter validation; true prospective clinical testing was rare (1.4%), and only a small subset of models had been implemented in practice (3.4%). Diagnostic models frequently achieved very high technical accuracy (AUC 0.90–1.00 in constrained tasks), while prognostic models showed moderate-to-high performance (AUC 0.75–0.95). Explainability was underreported, only 24% used any form of saliency mapping, Grad-CAM or feature importance analysis.

**Conclusions:**

AI in orthopedic trauma surgery demonstrates strong technical feasibility but remains overwhelmingly at the proof-of-concept stage. The field is characterized by limited external validation, minimal prospective evidence, scarce explainability, and insufficient workflow integration, factors that collectively hinder clinical translation. To bridge the gap from laboratory performance to real-world impact, future research must emphasize multicenter datasets, rigorous external and prospective validation, explainable AI, and user-centered implementation studies. AI has the potential to augment, rather than replace, orthopedic trauma care, but its safe and effective adoption requires substantial methodological maturation.

**Graphical Abstract:**

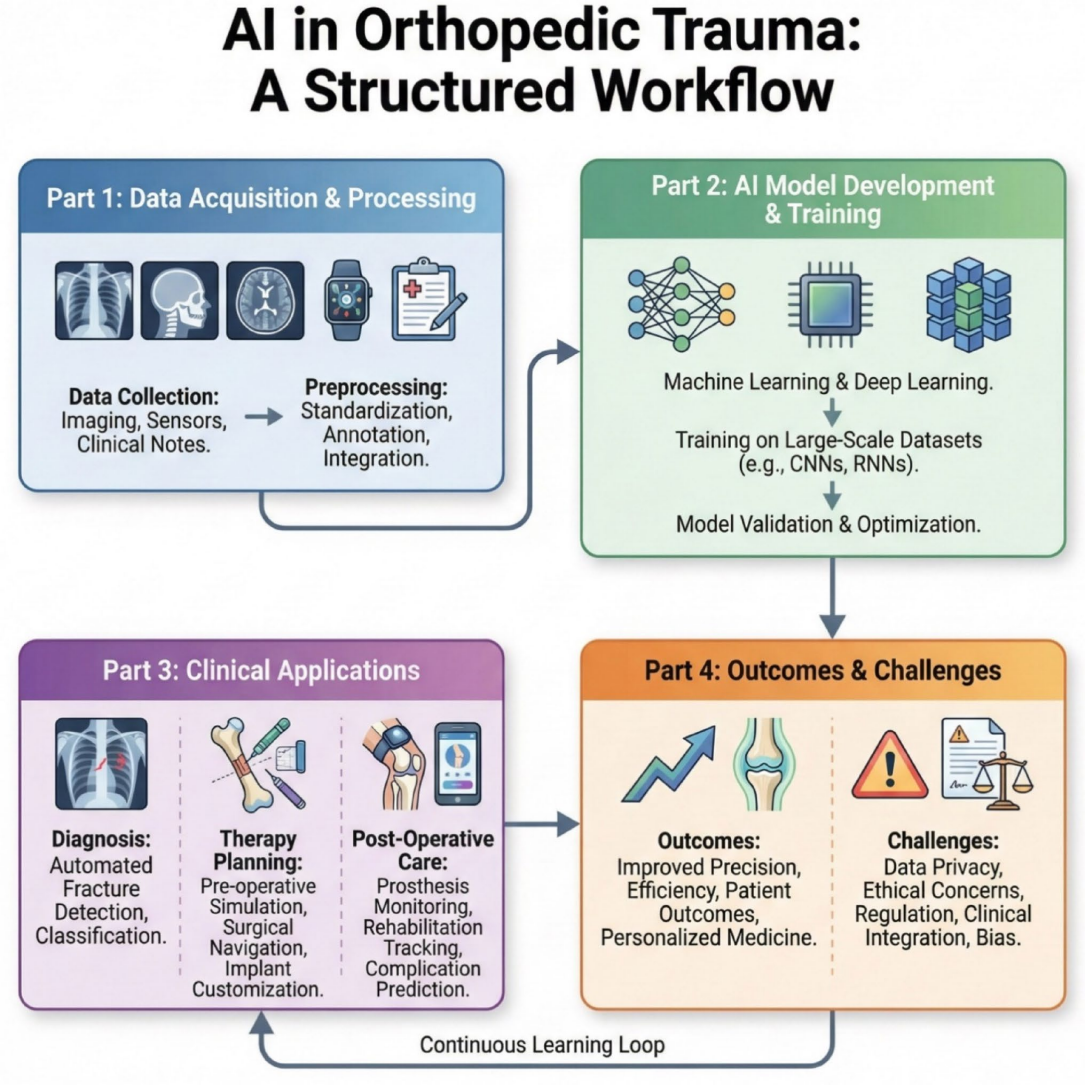

## Introduction

 Artificial intelligence (AI) and machine learning (ML) have rapidly evolved into influential drivers of innovation across medicine, supporting tasks ranging from advanced image interpretation to individualized outcome prediction and clinical decision support[1–3]. Within orthopedic trauma surgery, early investigations have shown that deep neural networks can detect fractures on radiographs with accuracy approaching that of expert clinicians[4, 5]. Likewise, machine learning based prognostic models have demonstrated superior performance over conventional risk scores in forecasting postoperative complications and trauma related mortality[6, 7]. Together, these developments underscore the potential of AI to enhance clinical decision-making by enabling faster diagnostic triage, more precise treatment stratification, and early identification of high-risk patients.

Despite these advances, the translation of AI from proof-of-concept models into routine clinical practice remains limited. Persistent concerns regarding data quality, model generalizability across diverse patient populations and the opacity of many AI architectures continue to impede widespread adoption[8]. Most published studies evaluate algorithmic performance retrospectively under idealized conditions, with relatively few conducting external validation or assessing real-world integration within heterogeneous clinical workflows[9]. Critical gaps include the scarcity of prospective trials demonstrating tangible patient benefit, limited regulatory clearance of AI applications in trauma care, and substantial challenges in embedding AI systems into healthcare information technology infrastructures[10].

Given the accelerating growth of research in this field, a scoping review is needed to systematically map current AI applications in orthopedic trauma surgery and to delineate unresolved challenges. While Mısır et al. (2025)[11] provided a comprehensive overview of publication volume, our review specifically focuses on the qualitative barriers to clinical implementation, namely explainability and prospective validation. This focus is crucial, as the translation of AI from proof-of-concept models into routine clinical practice remains limited due to persistent concerns regarding model transparency and real-world generalizability. This review synthesizes state-of-the-art developments across diagnostic imaging, injury classification, outcome prediction, surgical planning, and other emerging domains. Following the Preferred Reporting Items for Systematic Reviews and Meta-Analyses extension for Scoping Reviews (PRISMA-SCR) framework[12], we provide a comprehensive overview of the capabilities, limitations, and translational barriers of AI in orthopedic trauma, offering direction for future research and evidence-based implementation.

## Methods

We conducted a scoping review to map the current landscape of artificial intelligence (AI) applications in orthopedic trauma surgery, aiming for a structured and transparent approach aligned with PRISMA-SCR recommendations[12]. A broad search strategy was developed and executed using the AI research assistent Elicit in the Semantic Scholar, OpenAlex and PubMed corpora, which indexes both peer-reviewed journal articles and conference proceedings, covering all literature up to 2025. A 2025 systematic review of 23 comparative studies found that multidisciplinary databases like OpenAlex achieve broader coverage than traditional subscription databases[13]. The query incorporated key AI terms in combination with orthopedic trauma descriptors. We inputted the following exact Boolean string into Elicit’s search bar: (“artificial intelligence” OR “machine learning” OR “deep learning”) AND (“orthopedic trauma” OR “fracture” OR “trauma surgery” OR “musculoskeletal injury”) AND (diagnosis OR classification OR treatment OR surgery OR “outcome prediction”). We executed our search strategy directly within the Elicit interface rather than performing individual API calls or native manual searches across separate databases.

Large language models (LLMs, in this case OpenAI ChatGPT-4o) were used to accelerate certain components of the workflow, such as literature organisation and refinement of phrasing, without influencing interpretation of results[14–16]. All identified references underwent title and abstract screening for relevance to AI applications in traumatic orthopedic contexts. We included original studies of any design that applied machine learning or deep learning techniques to diagnostic, classification, treatment-support, or prognostic tasks in orthopedic trauma. Exclusion criteria comprised non-English publications, non-traumatic orthopedic conditions, animal or cadaver studies and non-original articles (reviews, editorials, and conference abstracts without primary data). To process the results, we utilized Elicit’s built-in filtering parameters to restrict records to original research involving our specified inclusion criteria. We screened the top 500 results returned by the Elicit relevance algorithm, establishing a stopping rule to halt screening when the relevance of the retrieved literature significantly diminished. Because Elicit queries a unified corpus, standard deduplication across different databases was largely handled by the platform itself. However, we exported the results and performed a final deduplication check using the reference management software Zotero, resulting in a final corpus of 497 unique records for abstract screening.

To ensure methodological integrity, human oversight was maintained at every stage. While Elicit was used to populate the preliminary data matrix, AI-suggested records underwent title, abstract and full-text screening by human reviewer against the predefined eligibility criteria. Furthermore, extracted data points, performance metrics and citations were manually verified against the original published full texts.

### Selection criteria

Studies were eligible if they met all of the following criteria:

(i) included human subjects with traumatic orthopedic injuries;
(ii) employed machine learning or deep learning methodologies for diagnosis, classification, treatment planning, or outcome prediction;
(iii) reported quantitative model performance metrics (e.g., accuracy, sensitivity, AUC);
(iv) involved a minimum sample size of 10 patients, cases, or images; and.
(v) were published in English in peer-reviewed journals or conference proceedings.

Studies focused on degenerative or metabolic orthopedic conditions were excluded.

### Data extraction and synthesis

Following the initial AI-assisted retrieval, title/abstract screening and subsequent full-text reviews of all eligible studies were conducted independently by a single primary reviewer. Because the final screening and data extraction were performed by one human reviewer, formal inter-rater disagreement resolution was not required; however, all extracted data points were manually cross-verified against the original source full texts to ensure accuracy. Data were extracted into a standardized charting template capturing: publication year, country, study design, population characteristics, injury type, AI methodology, application domain, data source, performance metrics, validation strategy, explainability, and limitations. To ensure consistency in our reported proportions, we applied the following operational definitions for key labels:

(i) External Validation: The AI model was tested on a completely independent dataset originating from a different hospital, institution, or geographic region than the training data, or on a distinct, recognized public dataset not used during model development.
(ii) Internal Validation: The AI model was tested using data from the same institution/source as the training data (e.g., temporal split, hold-out test set, or cross-validation).
(iii) Prospective Evaluation: The AI tool was evaluated in a forward-looking clinical setting where predictions or classifications were generated and recorded before the actual clinical outcomes or ground-truth diagnoses were known to the evaluating clinicians.
(iv) Implemented in Practice: The study explicitly reports that the AI model has been integrated into routine clinical workflow and is actively being used to guide or assist patient care outside of a purely experimental setup.
(v) Explainability (XAI): The study incorporates specific techniques (e.g., saliency maps, Grad-CAM, feature importance charts) designed to make the AI model’s decision-making process understandable to human clinicians.
(vi) We acknowledge the limitation that not all studies provide this information in a consistent manner. To increase accuracy, in the case of doubt, inconsistency or missing information, the regarding studies were excluded in this case.

**Fig. 1:**
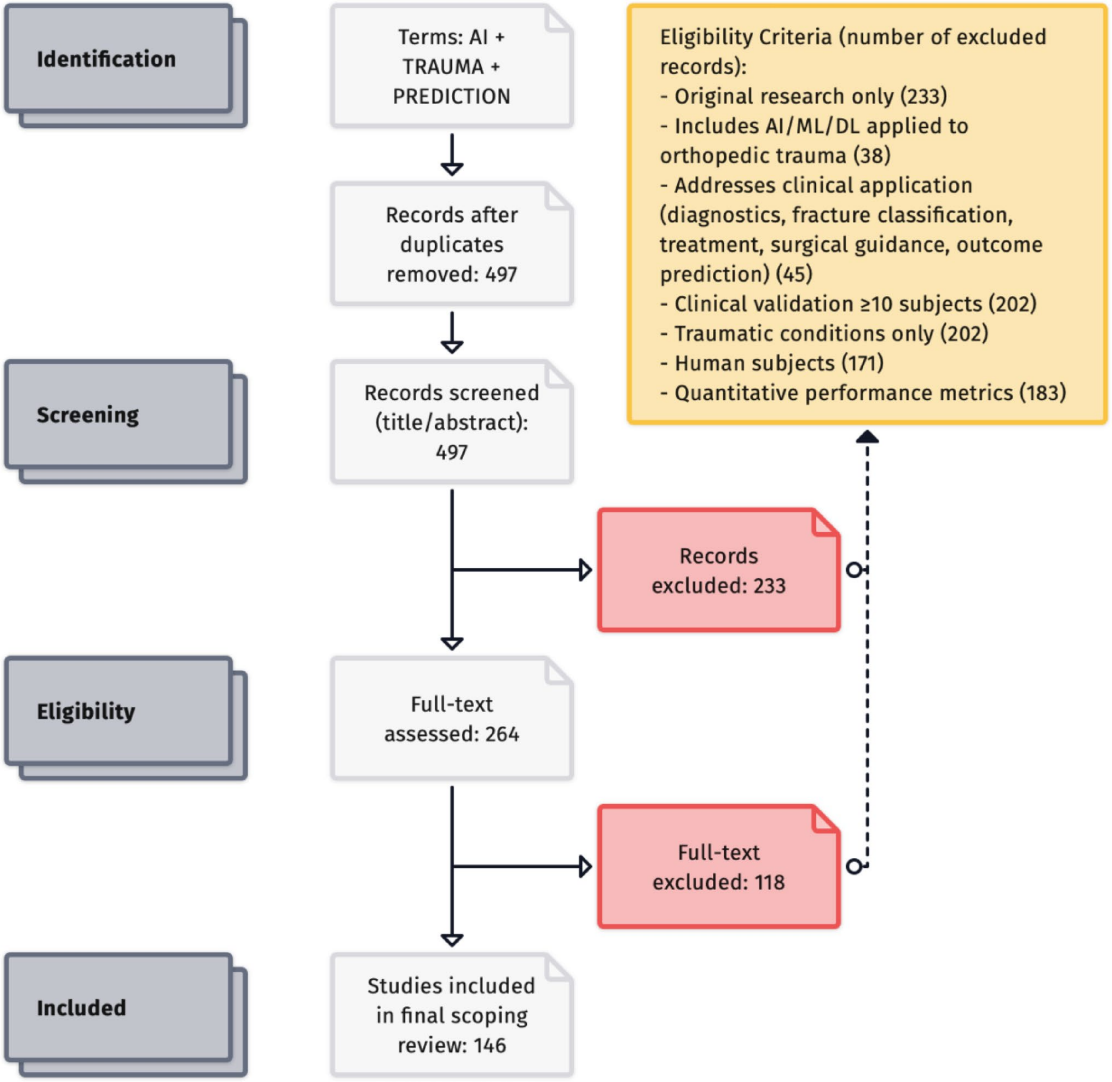
PRISMA-SCR flow diagram of study selection. A total of 497 records were identified through database searching. After screening titles and abstracts, 351 records were excluded for not meeting inclusion criteria (such as wrong topic, no AI component, or non-trauma focus). The remaining 146 studies were included in the qualitative synthesis for this scoping review. This flow diagram illustrates the study identification and screening process following PRISMA guidelines

Given the heterogeneity of study aims, datasets, and evaluation metrics, no meta-analysis was performed. Instead, findings were synthesized descriptively. We present trends in publication volume, geographical distribution, AI use cases, validation methods, performance ranges, and recurrently reported research gaps using narrative summaries, tables, and figures. Consistent with scoping review methodology, we aimed to characterize the breadth of existing work and identify areas requiring further investigation rather than performing formal quality appraisal of individual studies. The full workflow is visible in Fig. 1. The full list of studies can be found in the appendix.

## Results

### Literature search and selection

The search strategy yielded 497 unique records. After title/abstract screening, 146 studies met the inclusion criteria for this scoping review (Fig. 1). The majority of exclusions (351 records) were due to studies not meeting the inclusion criteria, most commonly because they were unrelated to orthopedic trauma (e.g. focused on degenerative conditions or non-orthopedic injuries), did not use any AI methods, were non-English, or were review/commentary pieces without original data. No additional eligible studies were identified through reference cross-checking beyond those found via the AI-augmented search. All 146 included studies underwent full-text data extraction and qualitative synthesis.

### Study characteristics

**Fig. 2:**
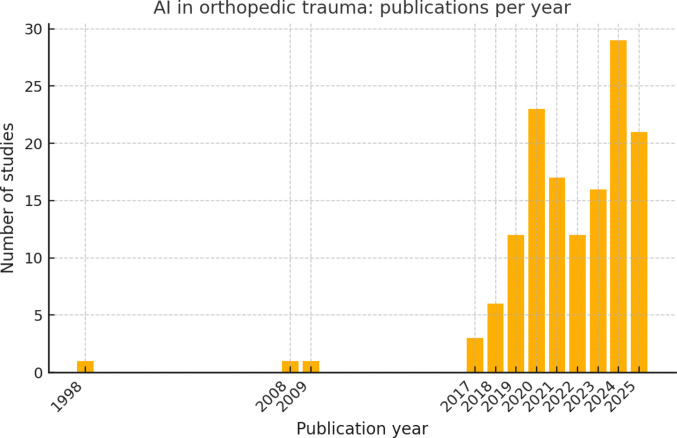
Annual publication output on AI in orthopedic trauma (1998–2025). Temporal trend of published studies demonstrating minimal activity before 2017, followed by a rapid increase in research output from 2018 onward, peaking between 2021 and 2024 with sustained high publication numbers in 2025

The 146 included studies were published between 1998 and 2025, with a marked increase in research activity in recent years. Only one study was published in the late 1990 s (1998) and only a handful appeared in the 2000 s, followed by a sharp uptick from 2017 onward. Over half of all included studies were published in 2020 or later, with 2024 alone accounting for 29 studies the highest annual count in our dataset. This surge in publications corresponds with the wider availability of deep learning methods and larger datasets in the late 2010s[17, 18]. Geographically, research contributions have come from around the world, though they are skewed toward certain regions (Fig. 2). The United States produced the highest number of studies (~16% of the total, 24 out of 146), followed by notable contributions from countries such as South Korea, Sweden, China, Australia, and the United Kingdom (each contributing several studies). Many other countries were represented by only one or two studies. It should be noted, however, that in nearly half of the publications the country of the study was not explicitly stated in the abstract (likely because the study used multi-center or international databases without a single country focus or didn’t mention the source).

In terms of study design, the vast majority of included studies were retrospective in nature. We identified only two studies that were prospective investigations (both were preliminary observational cohort studies), and there were no randomized controlled trials of AI interventions in orthopedic trauma to date. Most studies were single-center projects that trained and tested AI models on data from one hospital or institution. Only around 15–20% of studies performed any form of external validation using data from a different hospital or an external dataset, indicating that multicenter studies were relatively uncommon. Table 1 summarizes key characteristics of the included studies.

**Table 1:** Study Characteristics of Included Studies (*N* = 146)

Characteristic	Summary
Publication years	1998–2025 (over 52% of studies published since 2022; peak output in 2024 with 29 studies).
Countries (top contributors)	United States (24 studies, ~ 16%); South Korea (~5%); Sweden (4%); China (2%); Australia (2%); United Kingdom (2%); numerous other countries with 1–2 studies each.
Study designs	Predominantly retrospective observational studies (~99%); only 2 prospective studies (1%); no randomized trials to date.
Single vs. multicenter	~ 69% single-center studies including ~16% unclear or not stated; ~15% explicitly multi-center or external validation.
Data sources	Imaging data used in ~70% of studies (mostly X-ray radiographs; some CT/MRI); clinical registry or electronic health record (EHR) data used in ~30% (especially for outcome prediction).
Sample sizes	Varied widely. Imaging studies often included hundreds to thousands of radiographs (more than 100k images in one study); clinical studies ranged from tens to thousands of patients. Many studies utilized public datasets or national trauma databases for large sample sizes.

### Al applications

The applications of AI in orthopedic trauma spanned several major domains (Tables 2, 3). By far the most common focus was on medical imaging tasks, in particular, fracture detection and classification. A total of 37 studies (approximately 25% of the 146) addressed automated fracture detection (e.g. identifying the presence of a fracture on an X-ray or CT scan), and 20 studies (~14%) addressed fracture classification (such as classifying fracture type or severity on imaging). These imaging-related diagnostic tasks (often employing radiographs) together constituted a large portion of the research activity. Many of these works reported that AI models could achieve diagnostic accuracy comparable to orthopedic surgeons or radiologists for specific fracture types. For example, one deep learning model for proximal humerus fractures achieved 96% accuracy with an area under the ROC curve (AUC) of 1.00 in distinguishing fractures from normal cases. In that study, the Al’s performance was on par with fellowship-trained shoulder specialists and exceeded that of general orthopedic surgeons[19]. Another study documented near-perfect performance in automated hip fracture classification, with classification accuracy approaching 99% and AUC values in the 0.99–1.00.99.00 range for certain fracture categories[20]. However, it is important to note that such high performances were often reported on retrospective test datasets and for well-defined tasks; real-world performance may be lower due to more variable data and clinical complexity.

Aside from imaging, predictive modeling of clinical outcomes was the next major application area. Sixty-four studies (~44%) developed AI models to predict outcomes such as mortality, postoperative complications, or length of hospital stay in trauma patients. These prognostic studies typically applied machine learning to clinical datasets (e.g. trauma registries or electronic health records). Common targets included early mortality after injury, wound infection risk, need for intensive care or reoperation and functional outcomes. Generally, the machine learning models (often gradient boosting machines, random forests, or neural networks) showed moderate-to-high discriminative ability, with reported AUC values often in the 0.75–0.95 range for outcome prediction tasks. In several cases, the ML models outperformed traditional clinical risk scores or regression models; for instance, one study’s ML algorithm improved the AUC for trauma mortality prediction to 0.974[21]. However, improvements over existing scoring systems were not universal, and some authors noted only marginal gains.

A few other applications of AI in orthopedic trauma were identified, though these were relatively uncommon. For example, segmentation and quantitative image analysis were addressed in a small number of studies, e.g., one study used a deep learning model to automatically measure hematoma volume on pelvic trauma CT scans, and another monitored bone healing progress on serial radiographs using Al[22, 23]. Each of these topics was reported by only 1–2 studies (≤ 1% each). Notably, treatment planning and surgical guidance applications of AI, such as AI-driven decision support for selecting operative versus nonoperative management, surgical approach recommendation or preoperative virtual reduction, are rare but emerging in the literature[24]. A few recent studies have begun exploring AI for resource allocation and triage in trauma systems, but these were in early stages. While AI-driven preoperative templating is increasingly established in elective arthroplasty, our review confirms a distinct scarcity of such applications in orthopedic trauma. However, a small subset of included studies has begun to address this gap. Despite these early proof-of-concept examples, comprehensive clinical decision support tools and real-time intraoperative guidance remain highly under-explored.Unlike the predictable anatomy of degenerative joints, traumatic injuries present with highly variable fracture patterns, comminution, and fragment displacement, which pose significant challenges for current automated planning algorithms and explain the paucity of studies in this specific domain.

**Table 2:** AI Application Domains and Validation in Included Studies

Application Domain	Number of Studies (non-exclusive, % of 146)
Outcome prediction	64 (43.8)
Fracture detection	37 (25.3)
Fracture detection + classification	23 (15.8)
Fracture classification	20 (13.7)
Other/mixed	2 (1.4)
Internal validation only (single-center)	124 (85)
External validation (multi-center)	22 (15)
Prospective clinical evaluation	2 (1.4)

### Al techniques and model development

**Fig. 3:**
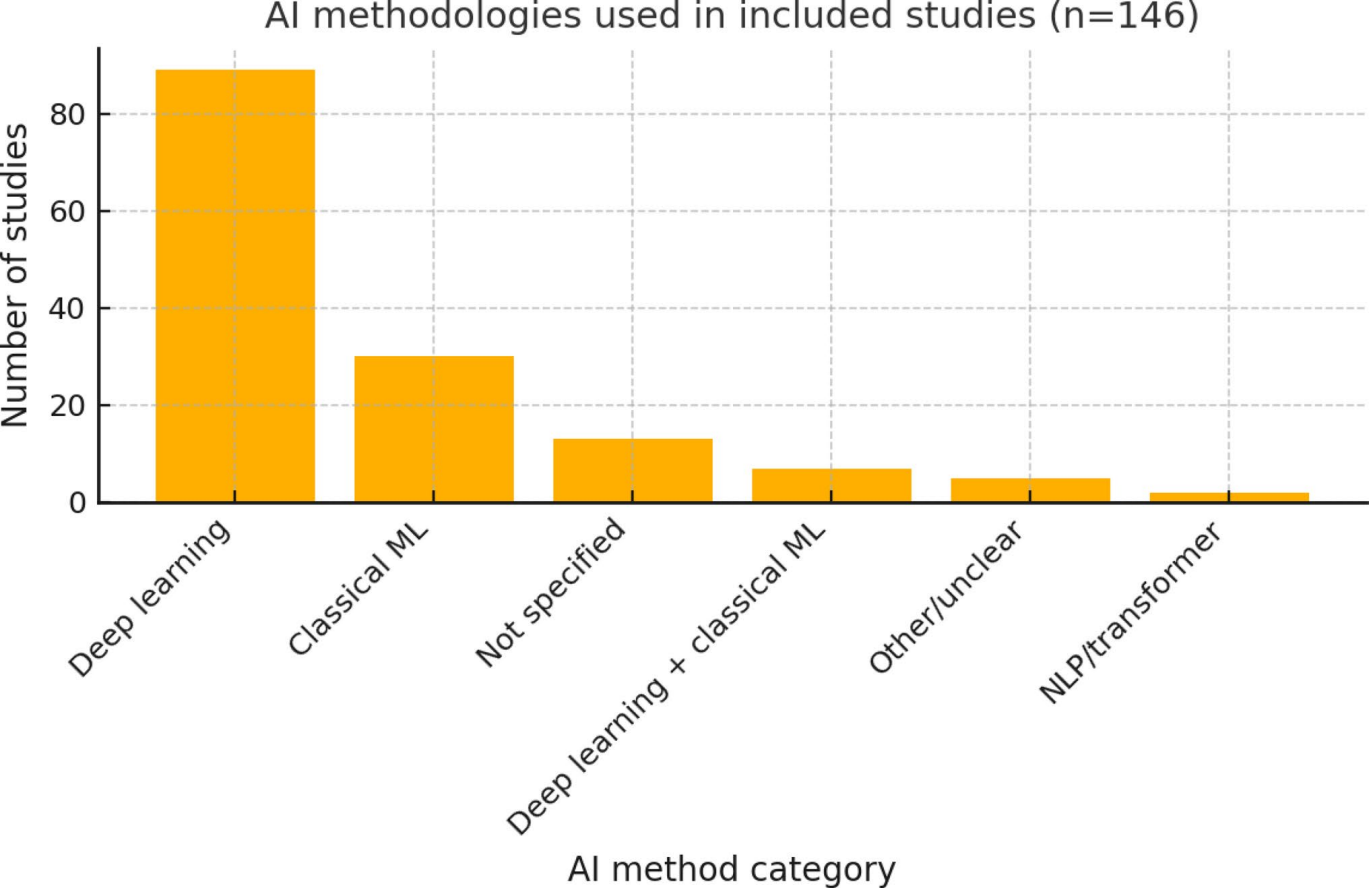
AI methodologies used in the included studies (*n* = 146). Bar chart showing the frequency of AI method categories across all studies. Deep learning dominates, followed by classical machine learning, studies without a specified method, hybrid DL + ML models, other/unclear approaches, and rare use of NLP/transformer architectures

**Fig. 4:**
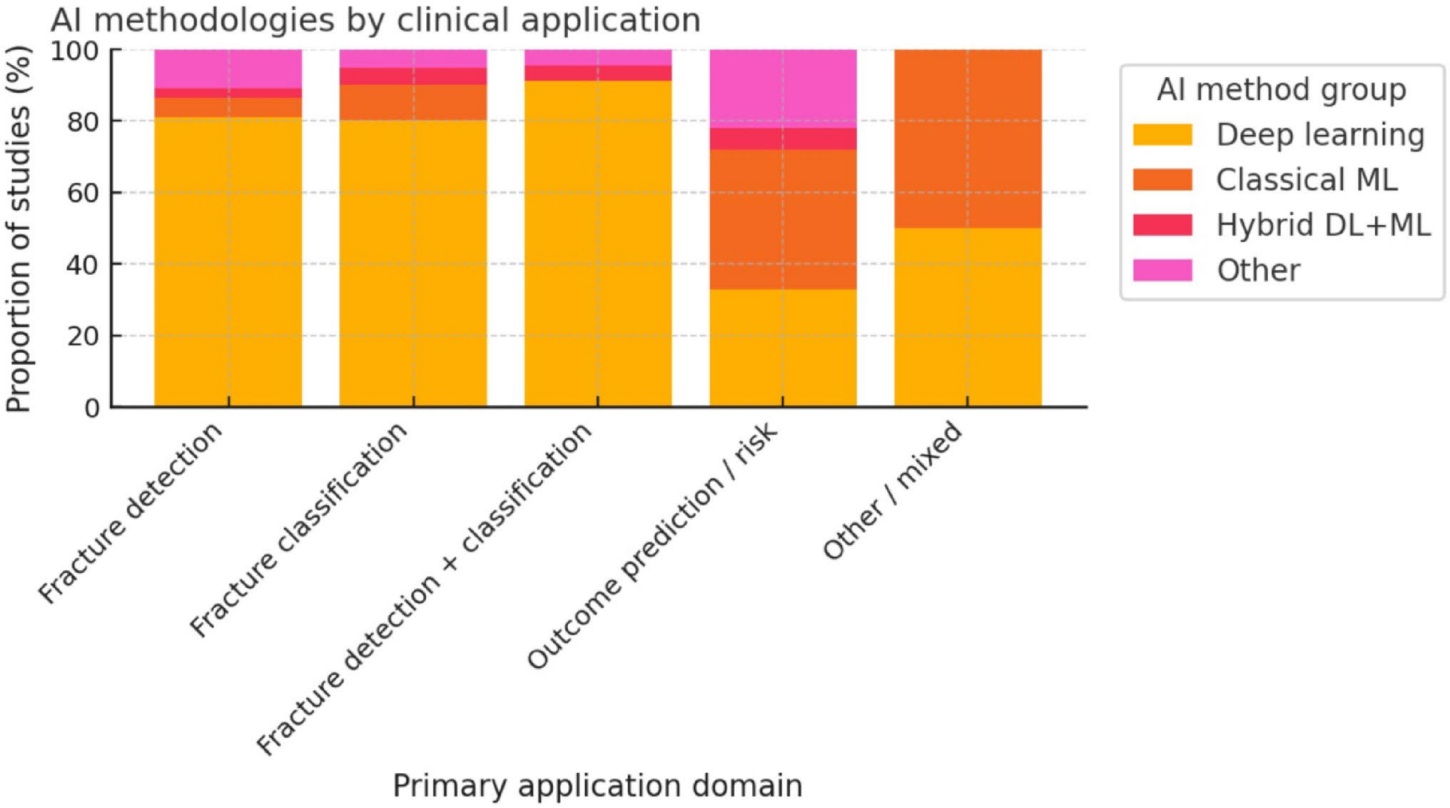
Distribution of AI methodologies across clinical application domains in orthopedic trauma. Stacked bar chart illustrating the proportional use of deep learning, classical machine learning, hybrid DL + ML approaches, and other methods across five primary domains: fracture detection, fracture classification, combined detection/classification, outcome prediction or risk stratification, and mixed/other applications

A wide range of AI and machine learning techniques were employed across the studies, reflecting advances over time (Fig. 3). Deep learning methods, particularly convolutional neural networks (CNNs) for image analysis, were the most prevalent approach, used in approximately 50–60% of studies overall (and the vast majority of imaging studies). Frequently mentioned CNN architectures included ResNet (in various studies on fracture detection), DenseNet, VGG, Xception, U-Net (for segmentation), and object detection models like YOLO. For instance, two studies applied the YOLO family of models for fracture detection on radiographs[25, 26], and several others fine-tuned popular CNNs (ResNet, InceptionV3, etc.) for classification tasks. Traditional machine learning algorithms (such as logistic regression, support vector machines, random forests, and gradient boosting machines) were also utilized, particularly in outcome prediction studies using tabular clinical data. A small number of works (~2) explored natural language processing (NLP) approaches, for example, using a BERT-based model to analyze clinical notes for injury coding. Overall, the choice of model was closely tied to the data type (Fig. 4): CNNs dominated in image-focused studies, whereas structured clinical data often saw tree-based ensembles or neural networks. In a few cases, ensemble models were built that combined multiple algorithms for improved performance.

### Model validation and performance

Model validation strategies were reported heterogeneously. Roughly 85% of studies relied on internal validation only, typically by holding out a test set from the same source or using cross-validation. Only 22 studies (15%) included any form of external or independent validation, as noted above. Moreover, the stage of translation of most AI models was still at the proof-of-concept or retrospective evaluation phase. Only two studies were designed as prospective clinical evaluations of an AI tool, and none was a randomized trial. Both demonstrated the potential of AI but underscored practical integration issues.

Across the included studies, reported performance metrics varied significantly depending on the task, data source, and crucially, the validation strategy. When evaluating models strictly within internal, retrospective hold-out sets (which comprised ~85% of the literature), technical performance was generally very high. For fracture detection and classification tasks on imaging, internally validated models frequently reported accuracies in the high 80 s to 90 s (%) and AUC values in the 0.90–1.00 range. Sensitivities and specificities above 90% were commonly achieved by top-performing models in these constrained tasks, sometimes rivaling the performance of orthopedic specialists in idealized, controlled comparisons.

However, these ‘near-perfect’ metrics must be interpreted strictly as constrained, retrospective proofs-of-concept. In the minority of studies (~15%) that clearly performed external or multicenter validation, performance metrics often showed greater variability or slight degradation, reflecting the challenges of generalizing across diverse patient populations and scanner hardware. Because true prospective clinical testing remains exceptionally rare (1.4%), there is currently insufficient evidence to confirm that the peak technical accuracies reported in internal datasets translate to equivalent real-world clinical performance.

For outcome prediction models, performance was more modest: AUCs in the 0.75–0.85 range were common for predicting outcomes like mortality or infection, though a few models exceeded 0.90 AUC for certain predictions (Table 4). Notably, in head-to-head comparisons, machine learning models often outperformed traditional risk stratification methods. However, the advantage of complex ML models over simpler logistic regression was not always substantial for every outcome, highlighting the need for careful evaluation of clinical added value.

**Table 3:** Summary of AI Performance Metrics Reported Across Studies

Performance Metric	Studies Reporting (*n*, %)	Range of Reported Values
Area Under ROC Curve (AUC)	78 (~53%)	0.75–1.00 (median ~0.90)
Accuracy	74 (~51%)	69% − 99.5%
Sensitivity	48 (~33%)	70% − 100%
Specificity	49 (~34%)	70% − 100%
Precision (Positive Predictive Value)	35 (~24%)	80% − 99%
F1-score	19 (~13%)	0.80–0.99

**Table 4:** Summary of Studies Monitoring Patient Data

Study	Condition	Outcome	AI Performance	Clinical Validation
Han et al., 2024	Critically ill orthopaedic trauma patients	30-day mortality	AUC: 0.974 (eXGBM)	Internal and external
Machine Learning Consortium, 2021	Tibial shaft fractures	Infection	AUC: 0.75–0.81	Internal
Dreizin et al., 2020	Pelvic fractures	Need for interventions, mortality	AUC: 0.83 (interventions)	Internal
Badgeley et al., 2018	Hip fractures	Fracture prediction	AUC: 0.78–0.91	Internal
Lex et al., 2023	Hip fractures	Postoperative outcomes	AUC: 0.80 (30-day mortality), 0.81 (1-year mortality)	Internal
Lim et al., 2025	Distal radius fractures	Need for surgery	Accuracy: 92.98%	Internal
Teng et al., 2023	Post-fracture surgery	Bone healing	Accuracy: 0.73, AUC: 0.67–0.90	Internal and external
Hendrickx et al., 2020	Tibial shaft fractures	PMF risk	AUC: 0.89	Internal
Hertz et al., 2019	Pelvic fractures	Bladder rupture	Accuracy: 97.8%, AUC: 0.99	Internal
Staziaski et al., 2021	Torso trauma	LOS, ICU admission	AUC: up to 0.87	Internal
Dapnall et al., 2021	Wrist fractures	Fracture outcomes	No mention found	Internal

In summary, AI systems in orthopedic trauma have demonstrated strong technical performance in experimental settings, particularly for image-based tasks like fracture detection. In numerous studies, AI models achieved performance on par with, or even exceeding, that of human clinicians in specific diagnostic tasks. For outcome predictions, AI has shown moderate improvements over conventional risk models in some cases. Additionally, few studies so far have incorporated explainability features or user-centered design considerations, only a handful of papers mentioned using techniques like saliency maps or feature importance to provide clinicians with interpretable outputs from the Al. The paucity of explainability and usability testing suggests that many AI models are still viewed as research prototypes rather than ready-for-clinic tools.

## Discussion

This scoping review reveals a field in rapid development, with AI applications in orthopedic trauma expanding dramatically in the past five to ten years. The findings illustrate that AI techniques, especially deep learning for image analysis, have achieved impressive results in detecting and classifying fractures, as well as promising performance in outcome prediction models. Fracture imaging (diagnosis and classification) and prognostic risk modeling currently dominate the landscape of published studies, together accounting for the majority of applications explored. In these domains, numerous algorithms have demonstrated technical feasibility: For example, CNN-based models can identify fractures on X-rays with accuracies that approach those of subspecialist radiologists, and machine learning predictors can modestly improve risk stratification for trauma patients over traditional scoring systems[27–29]. These successes indicate substantial potential for AI to augment clinical care, by aiding in faster and more accurate diagnosis, flagging high-risk patients, and possibly reducing human error in trauma management.

While AI models demonstrate strong technical feasibility, reaching AUCs of 0.90–1.00 in specific diagnostic tasks, we must emphasize that these successes represent a highly constrained, retrospective proof-of-concept stage of development. The current evidence base is overwhelmingly dominated by retrospective study designs and internal validation strategies. Consequently, the headline performance metrics reported in much of the literature do not yet reflect mature clinical readiness. Realizing the potential of AI in routine orthopedic trauma practice requires a fundamental shift in research methodology. The field must transition from prioritizing near-perfect internal accuracy toward conducting robust external validations across diverse patient populations and prioritizing prospective clinical trials, which are strictly necessary to establish true real-world generalizability and effectiveness. In our review, only about 15% of studies tested their models on external data, and only two conducted prospective evaluations and notably, no randomized controlled trial has yet examined an AI intervention in this field. This highlights that while technical research is proliferating, translational research and implementation of technology is lagging. Future studies should emphasize generalizability of AI models, most important by incorporating multi-center data, testing algorithms on populations from different regions or healthcare systems and assessing performance on cases from outside the original training distribution. Such efforts are critical to ensure that these models maintain their accuracy and utility outside of the development environment.

Additionally, there is a need to move beyond pure performance metrics and address clinical integration and user acceptability. Almost none of the studies deeply explored how an AI tool would fit into clinical workflows or how clinicians would interact with it. Issues of explainability and interpretability are paramount[30]. Surgeons and frontline providers must be able to understand and trust AI recommendations in order to incorporate them into decision-making. Only a very small subset of the reviewed studies included any form of explainable AI technique (such as heatmaps highlighting fracture locations, or feature importance for predictions) and none formally evaluated the impact of AI explanations on clinician decision confidence. Explainability will have crucial meaning for legal actions and understanding actions. Going forward, incorporating explainable AI (XAI) features and designing user-friendly interfaces will be crucial for successful adoption. For example, providing heatmap overlays on radiographs to show why an algorithm believes a fracture is present, or giving clinicians a ranked list of risk factors driving a mortality prediction, could help build confidence in AI systems.

We also observed that prospective implementation studies are extremely scarce but beginning to emerge. Such interventional studies are critical for understanding the real-world impact of AI tools, including workflow integration challenges, changes in diagnostic or treatment decision metrics and any unintended consequences. The results of that study indicated that AI assistance significantly increased the sensitivity of fracture detection by emergency clinicians (with a notable benefit for those with less musculoskeletal training) underscoring that AI can serve as a useful adjunct. However, it also highlights the need to assess which clinicians benefit most and under what conditions. Future research should include pilot deployments of AI systems in trauma settings, accompanied by metrics on patient outcomes, efficiency, and user feedback, to fully elucidate the clinical value added by AI.

Another important consideration is data bias and ethical use of AI in orthopedic trauma. Trauma populations and injury patterns can differ substantially by region, age, sex, and other factors; if AI models are trained on unrepresentative datasets, they may exhibit biased performance (for example, missing injuries in under-represented groups or uncommon scenarios). Only a few studies in our review discussed bias or fairness and none did a formal bias analysis. As these tools advance toward clinical use, stakeholders will need to ensure that AI algorithms are trained on diverse, representative data to avoid perpetuating health disparities. Transparency in algorithm development and reporting (for instance, using checklists like TRIPOD-AI or CONSORT-AI for studies[31, 32]) will aid in this effort. Regulatory and legal considerations are also on the horizon. As of the time of this review and already mentioned, only few AI applications in orthopedic trauma have achieved regulatory clearance (e.g. FDA clearance in the United States) and limited market deployment. Navigating regulatory pathways will require rigorous evidence of safety and efficacy. Clear guidelines on liability, for example, determining responsibility when an AI influenced decision leads to an adverse outcome, have yet to be established in the orthopedic domain and underscore the strong need for XAI.

Despite these challenges, the outlook for AI in orthopedic trauma is optimistic. We are witnessing a dynamic and rapidly advancing field with considerable potential to augment clinical care (rather than replace the human element). The current applications are heavily centered on fracture imaging and prognostic modeling, which, as shown in this review, have achieved strong performance in experimental settings. Moving forward, the focus should shift toward validation in real-world environments and integration into clinical workflows in a manner that supports clinicians (the “augmented intelligence” paradigm). For instance, AI-driven diagnostic support might first be used as a secondary reader for fracture radiographs, flagging possibly missed injuries for radiologist review, or as a triage tool that alerts on high-risk patients to trauma teams. Such integration needs to be done thoughtfully, with training of end-users and monitoring of outcomes.

We also identify opportunities for future research directions. One is the exploration of AI for areas that have seen little attention, such as surgical decision support (e.g., recommending fixation vs. replacement in complex fractures) and rehabilitation/functional outcome prediction. Another is the development of multimodal AI models that combine imaging with clinical data, laboratory results, or wearable sensor data, enabling a more holistic assessment of the trauma patient. Our review found few truly multimodal studies so far, representing a frontier for innovation. Furthermore, continuing advances in techniques like federated learning or swarm AI could facilitate multi-center collaborations without compromising patient data privacy, thereby increasing the diversity of training data for models.

Finally, we acknowledge the methodological limitations associated with utilizing AI-assisted literature discovery tools (such as Elicit) and Large Language Models (LLMs) in the scoping review process. While these tools significantly accelerate literature retrieval, deduplication, and phrasing refinement, they also introduce potential blind spots. To mitigate this and ensure scientific rigor, rigorous human oversight was maintained throughout the process: AI-suggested records were screened by a human reviewer under the same strict eligibility criteria, all exclusions were verified.

In conclusion, our scoping review indicates that AI in orthopedic trauma surgery has made significant strides in technical development, particularly in diagnostic imaging and outcome prediction, but remains in an early phase regarding clinical translation. By addressing the gaps in validation, explainability, bias and integration, and by fostering multidisciplinary collaboration between data scientists, orthopedic surgeons, trauma specialists, and other stakeholders, the next few years could witness AI evolving from an exciting research concept to a reliable assistant in the orthopedic trauma suite. Ultimately, if these challenges are met, AI has the potential to enhance orthopedic trauma care by improving diagnostic accuracy, optimizing treatment decisions, and identifying high-risk patients for timely intervention, thereby improving patient outcomes and healthcare efficiency.

## Conclusion

Artificial intelligence applications in orthopedic trauma are growing rapidly and show great promise. In diagnostic tasks like fracture detection and classification, AI systems (especially deep learning models) have achieved accuracy levels approaching those of expert clinicians, suggesting a role as valuable diagnostic aids. In prognostic modeling, machine learning algorithms can outperform some traditional risk scores in predicting outcomes such as mortality and complications, pointing toward improved patient stratification. However, most studies to date have been retrospective and single-center, and true clinical impact has yet to be proven. There remains a substantial gap between AI model development and its implementation in clinical practice. Key steps moving forward include extensive external validation of models (or multicenter swarm learning), incorporation of explainability to build clinician trust and build a legal foundation and prospective trials or pilot deployments to demonstrate efficacy and safety in real-world settings. Few AI tools in this domain have so far reached regulatory approval or routine use, but ongoing research and innovation are rapidly addressing issues of generalizability and integration.

In summary, AI stands poised to augment orthopedic trauma care by assisting clinicians, improving the speed and accuracy of fracture diagnosis, supporting decision-making, and enhancing outcome predictions, but its successful adoption will depend on closing current research gaps and ensuring these technologies are deployed responsibly, equitably and in harmony with clinical workflows. With continued interdisciplinary collaboration and rigorous validation, AI has the potential to become a reliable adjunct in the orthopedic trauma surgeon’s armamentarium, ultimately improving patient care in this challenging field.

## Data Availability

The data that support the findings of this study are available from the corresponding author upon reasonable request.

## Appendix: Full list of included studies (primary literature)

1. Aedo Lopez M, Castro Gutierrez EG. Implementación de un Modelo basado en técnicas de Deep Learning aplicado a la Visión Computacional en la Clasificación de Imágenes de Rayos X, para el soporte del diagnóstico de lesiones traumatológicas de la Estructura Pélvica. 2019: Latin American and Caribbean Consortium of Engineering Institutions.
2. Agarwal R, Singh J, Gupta V. Application of machine learning in rotary ultrasonic-assisted orthopedic bone drilling: A biomechanical pull out in vitro study. *Proceedings of the Institution of Mechanical Engineers, Part C: Journal of Mechanical Engineering Science* 2024; **238**(23): 10911-21.
3. Agarwalla A, Gowd A, Cody EA, Peterson A, Tan EW, Liu J. Prediction of Short-Term Postoperative Complications Following Open Reduction Internal Fixation of Acute Ankle Fractures. *Foot & Ankle Orthopaedics* 2024; **9**(2).
4. Agarwalla A, Lu Y, Reinholz AK, Marigi EM, Liu JN, Sanchez-Sotelo J. Identifying clinically meaningful subgroups following open reduction and internal fixation for proximal humerus fractures: a risk stratification analysis for mortality and 30-day complications using machine learning. *JSES International* 2024; **8**(5): 932 − 40.
5. Akbarian E, Mohammadi M, Tiala E, et al. Development and validation of an artificial intelligence model for the classification of hip fractures using the AO-OTA framework. *Acta Orthopaedica* 2024; **95**: 340-7.
6. Almaghrabi F, Xu D-L, Yang J-B. An application of the evidential reasoning rule to predict outcomes following traumatic injuries. 2020/8/13/: WORLD SCIENTIFIC. p. 849 − 56.
7. Anderson PG, Baum GL, Keathley N, et al. Deep Learning Assistance Closes the Accuracy Gap in Fracture Detection Across Clinician Types. *Clin Orthop Relat Res* 2022; **481**(3): 580-8.
8. Atkinson CJ, Seth I, Seifman MA, Rozen WM, Cuomo R. Enhancing Hand Fracture Care: A Prospective Study of Artificial Intelligence Application With ChatGPT. *Journal of Hand Surgery Global Online* 2024; **6**(4): 524-8.
9. Axenhus M, Wallin A, Havela J, et al. Automated diagnosis and classification of metacarpal and phalangeal fractures using a convolutional neural network: a retrospective data analysis study. *Acta Orthopaedica* 2025; **96**.
10. Azarpira M, Belhadj I, Khodja M. Using Deep Learning to Suggest Treatment for Proximal Humerus Fractures. Studies in Health Technology and Informatics: IOS Press; 2024.
11. Badgeley MA, Zech JR, Oakden-Rayner L, et al. Deep learning predicts hip fracture using confounding patient and healthcare variables. *npj Digital Medicine* 2019; **2**(1).
12. Beri M, Singh Gill K, Upadhyay D, Devliyal S. Fracture Forecasting through Deep Learning's Role in Bone Injury Detection and Classification. 2024/7/27/: IEEE. p. 969 − 74.
13. Bhuiyan SS, Tarin LT, Niaz MS, Dolon MN, Afroz A, Rahman R. Fracture Classification in Musculoskeletal Radiographs Using Custom CNN and Ensemble Learning. 2024/5/2/: IEEE. p. 764-9.
14. Bolourani S, Thompson D, Siskind S, Kalyon BD, Patel VM, Mussa FF. Cleaning Up the MESS: Can Machine Learning Be Used to Predict Lower Extremity Amputation after Trauma-Associated Arterial Injury? *Journal of the American College of Surgeons* 2021; **232**(1): 102-13e4.
15. Bonnin M, Müller-Fouarge F, Estienne T, et al. Artificial intelligence assisted assessment of pre- and post-arthroplasty lower limb alignment on long-leg and knee close-up X-rays. EasyChair. p. 8 − 2.
16. Braun BJ, Histing T, Menger MM, et al. Wearable activity data can predict functional recovery after musculoskeletal injury: Feasibility of a machine learning approach. *Injury* 2024; **55**(2): 111254.
17. Breu R, Avelar C, Bertalan Z, et al. Artificial intelligence in traumatology. *Bone Joint Res* 2024; **13**(10): 588 − 95.
18. Brink FW, Adler B, Bambach S, et al. Using deep learning for estimation of time-since-injury in pediatric accidental fractures. *Pediatr Radiol* 2025; **55**(6): 1257-69.
19. Buscarini L. Enhancing Patient Rehabilitation Outcomes: Artificial Intelligence-Driven Predictive Modeling for Home Discharge in Neurological and Orthopedic Conditions.
20. Cai Y, Jiang X, Dai W, Yu Q. In-hospital Mortality Prediction among Patients with Fractures of Pelvis and Acetabulum in Intensive Care Unit: Machine Learning versus Conventional System. *Research Square* 2021.
21. Cardosi JD, Shen H, Groner J, Armstrong M, Xiang H. Machine Intelligence for Outcome Predictions of Trauma Patients During Emergency Department Care. *arXivorg* 2020.
22. Cardosi JD, Shen H, Groner JI, Armstrong M, Xiang H. Machine learning for outcome predictions of patients with trauma during emergency department care. *BMJ Health Care Inform* 2021; **28**(1): e100407.
23. Carter M, McGaughey T, Bardes J, Khodaverdi M, Adler N. 17 Optimizing trauma prognostication via machine learning: Automating frailty detection in geriatric trauma patients. *J Clin Trans Sci* 2025; **9**(s1): 6–7.
24. Chawhan L, Naik MM, Madhura R, Ramya V, Bhanushree KJ, Poorvitha HR. Bone Fracture Detection and Classification using Machine Learning. 2024/8/9/: IEEE. p. 1–8.
25. Cheng L-C, Liu C-F, Yeh C-C. “Could She/He Walk Out of the Hospital?”: Implementing AI Models for Recovery Prediction and Doctor-Patient Communication in Major Trauma. *Diagnostics* 2025; **15**(13): 1582.
26. Chiu IM, Lin C-HR, Yau F-FF, et al. Use of a Deep-Learning Algorithm to Guide Novices in Performing Focused Assessment With Sonography in Trauma. *JAMA Network Open* 2023; **6**(3): e235102.
27. Chung SW, Han SS, Lee JW, et al. Automated detection and classification of the proximal humerus fracture by using deep learning algorithm. *Acta Orthopaedica* 2018; **89**(4): 468 − 73.
28. Dandurand C, Schnake K, Oner FC, Dvorak M, Fallah N. 159. Predictive algorithm for surgery recommendation in thoracolumbar burst fractures without neurological deficits. *The Spine Journal* 2023; **23**(9): S81-S2.
29. DeBaun MR, Chavez G, Fithian A, et al. Artificial Neural Networks Predict 30-Day Mortality After Hip Fracture: Insights From Machine Learning. *J Am Acad Orthop Surg* 2020; **29**(22): 977 − 83.
30. Dipnall JF, Lu J, Gabbe BJ, et al. Comparison of state-of-the-art machine and deep learning algorithms to classify proximal humeral fractures using radiology text. *European Journal of Radiology* 2022; **153**: 110366.
31. Dipnall JF, Page R, Du L, et al. Predicting fracture outcomes from clinical registry data using artificial intelligence supplemented models for evidence-informed treatment (PRAISE) study protocol. *PLOS ONE* 2021; **16**(9): e0257361.
32. Doerr SA, Weber-Levine C, Hersh AM, et al. Automated prediction of the Thoracolumbar Injury Classification and Severity Score from CT using a novel deep learning algorithm. *Neurosurgical Focus* 2022; **52**(4): E5.
33. Dr Ruban S SSNKS, Abhishek K MMJ. HAND FRACTURE DETECTION USING DEEP LEARNING TECHNIQUES. *Redshi Arch* 2020; **2**.
34. Dreizin D, Goldmann F, LeBedis C, et al. An Automated Deep Learning Method for Tile AO/OTA Pelvic Fracture Severity Grading from Trauma whole-Body CT. *J Digit Imaging* 2021; **34**(1): 53–65.
35. Dreizin D, Zhou Y, Chen T, et al. Deep learning-based quantitative visualization and measurement of extraperitoneal hematoma volumes in patients with pelvic fractures: Potential role in personalized forecasting and decision support. *J Trauma Acute Care Surg* 2019; **88**(3): 425 − 33.
36. Esfandiari H, Andreß S, Herold M, Böcker W, Weidert S, Hodgson AJ. A Deep Learning Approach for Single Shot C-Arm Pose Estimation. 2020: EasyChair. p. 69 − 3.
37. Floyd SB, Almeldien AG, Smith DH, et al. Using artificial intelligence to develop a measure of orthopaedic treatment success from clinical notes. *Front Digit Health* 2025; **7**.
38. Gale W, Oakden-Rayner L, Carneiro G, Bradley A, Palmer L. Detecting hip fractures with radiologist-level performance using deep neural networks. *arXivorg* 2017.
39. Gao Y, Soh NYT, Liu N, et al. Application of a deep learning algorithm in the detection of hip fractures. *iScience* 2023; **26**(8): 107350.
40. Groot OQ, Bongers MER, Ogink PT, et al. Does Artificial Intelligence Outperform Natural Intelligence in Interpreting Musculoskeletal Radiological Studies? A Systematic Review. *Clin Orthop Relat Res* 2020; **478**(12): 2751-64.
41. Gupta P, Shen H-J, Patel K, Guo R, Heinz ER, Manyam R. Artificial intelligence for predicting 30-day mortality after surgery for femoral shaft fractures: A retrospective study. *Indian Journal of Anaesthesia* 2025; **69**(6): 606 − 14.
42. Gutierrez-Naranjo JM, Moreira A, Valero-Moreno E, Bullock TS, Ogden LA, Zelle BA. ­A machine learning model to predict surgical site infection after surgery of lower extremity fractures. *International Orthopaedics (SICOT)* 2024; **48**(7): 1887-96.
43. Guy S, Jacquet C, Tsenkoff D, Argenson J-N, Ollivier M. Deep learning for the radiographic diagnosis of proximal femur fractures: Limitations and programming issues. *Orthopaedics & Traumatology: Surgery & Research* 2021; **107**(2): 102837.
44. Han T, Xiong F, Sun B, Zhong L, Han Z, Lei M. Development and validation of an artificial intelligence mobile application for predicting 30-day mortality in critically ill patients with orthopaedic trauma. *International Journal of Medical Informatics* 2024; **184**: 105383.
45. He Q, Chen S, Li L. Image Enhancement Technology Based on Deep Trust Network Model in Clinical Treatment of Traumatology and Orthopedics. *Journal of Healthcare Engineering* 2021; **2021**: 1–11.
46. Hendrickx LAM, Sobol GL, Langerhuizen DWG, et al. A Machine Learning Algorithm to Predict the Probability of (Occult) Posterior Malleolar Fractures Associated With Tibial Shaft Fractures to Guide “Malleolus First” Fixation. *Journal of Orthopaedic Trauma* 2020; **34**(3): 131-8.
47. Hertz AM, Hertz NM, Johnsen NV. Identifying bladder rupture following traumatic pelvic fracture: A machine learning approach. *Injury* 2020; **51**(2): 334-9.
48. Inoue T, Maki S, Furuya T, et al. Automated fracture screening using an object detection algorithm on whole-body trauma computed tomography. *Scientific Reports* 2022; **12**(1).
49. Ito S, Nakashima H, Segi N, et al. A deep learning-based prediction model for prognosis of cervical spine injury: a Japanese multicenter survey. *European Spine Journal* 2025; **34**(9): 3714-23.
50. Jeon Y-D, Kang M-J, Kuh S-U, et al. Deep Learning Model Based on You Only Look Once Algorithm for Detection and Visualization of Fracture Areas in Three-Dimensional Skeletal Images. *Diagnostics* 2023; **14**(1): 11.
51. Jeon YD, Jung K-H, Kim M-S, Kim H, Yoon D-K, Park K-B. Clinical validation of artificial intelligence-based preoperative virtual reduction for Neer 3- or 4-part proximal humerus fractures. *BMC Musculoskeletal Disorders* 2024; **25**(1).
52. Ji S-Y, Najarian K, Smith R, Huynh T. COMPUTER AIDED TRAUMATIC PELVIC INJURY DECISION MAKING. 2008.
53. Ji S-Y, Smith R, Huynh T, Najarian K. BMC Medical Informatics and Decision Making. 2009.
54. Jiang W, Liu T, Sun B, et al. An artificial intelligence application to predict prolonged dependence on mechanical ventilation among patients with critical orthopaedic trauma: an establishment and validation study. *BMC Musculoskeletal Disorders* 2024; **25**(1).
55. Jiménez-Sánchez A, Kazi A, Albarqouni S, et al. Precise proximal femur fracture classification for interactive training and surgical planning. *Int J CARS* 2020; **15**(5): 847 − 57.
56. Jiménez-Sánchez A, Kazi A, Albarqouni S, et al. Towards an Interactive and Interpretable CAD System to Support Proximal Femur Fracture Classification. *arXivorg* 2019.
57. Jiménez-Sánchez A, Mateus D, Kirchhoff S, et al. Medical-based Deep Curriculum Learning for Improved Fracture Classification. Lecture Notes in Computer Science: Springer International Publishing; 2019: 694–702.
58. Jo SW, Khil EK, Lee SA, Lim J, Lee JH, Yoon YS. Deep learning system for automated detection of posterior ligamentous complex injury in patients with thoracolumbar fracture on magnetic resonance imaging. ISMRM.
59. Jones RM, Sharma A, Hotchkiss R, et al. Assessment of a deep-learning system for fracture detection in musculoskeletal radiographs. *npj Digital Medicine* 2020; **3**(1).
60. Joshi U, Raparthi M, Reddy R, Kumar P, Thuniki P, Shabaz M. Ultrasound-Guided Deep Learning for Regional Nerve Block Anesthesia in Scapular Fracture. 2024/4/26/: IEEE. p. 1–6.
61. Kandel I, Castelli M, Popovič A. Musculoskeletal Images Classification for Detection of Fractures Using Transfer Learning. *J Imaging* 2020; **6**(11): 127.
62. Kandel I, Castelli M, Popovič A. Comparing Stacking Ensemble Techniques to Improve Musculoskeletal Fracture Image Classification. *J Imaging* 2021; **7**(6): 100.
63. Kaur A, Singh Gill K, Rajput K, Singh V. New Era in Fracture Diagnosis using Deep Learning's Role in Precision Prediction. 2024/8/8/: IEEE. p. 830-4.
64. Kedija S. Machine learning technique in application and comparison in pediatric fracture healing time / Kedija Seid. 2018.
65. Kekatpure A, Kekatpure A, Deshpande S, Srivastava S. Development of a diagnostic support system for distal humerus fracture using artificial intelligence. *International Orthopaedics (SICOT)* 2024; **48**(5): 1303-11.
66. Khan K, Ali A, Haider A, Khan NA, Khan J. Investigation and classification of bone fracture using a deep learning model. *Asian J Sci Eng Technol* 2025; **4**(1): 54–68.
67. Khan MI, Pavithra M, Arthi M, Reshma M. Bone Fracture Identification with Deep Learning Model using Resnet50.
68. Killeen BD, Gao C, Oguine KJ, et al. An autonomous X-ray image acquisition and interpretation system for assisting percutaneous pelvic fracture fixation. *Int J CARS* 2023; **18**(7): 1201-8.
69. Kim DH, MacKinnon T. Artificial intelligence in fracture detection: transfer learning from deep convolutional neural networks. *Clinical Radiology* 2018; **73**(5): 439 − 45.
70. Kim H, Jeon YD, Park KB, et al. Automatic segmentation of inconstant fractured fragments for tibia/fibula from CT images using deep learning. *Scientific Reports* 2023; **13**(1).
71. Kim S, Koh HK, Lee H, Shin HJ. Deep-Learning Method for the Diagnosis and Classification of Orbital Blowout Fracture Based on Computed Tomography. *Journal of Oral and Maxillofacial Surgery* 2025; **83**(8): 980-9.
72. Klein C, Fondu P, Ghazali DA, Rotari V, Abou-Arab O, David E. Artificial intelligence and human expertise in hand trauma diagnosis: A collaborative approach. *Orthopaedics & Traumatology: Surgery & Research* 2025: 104338.
73. Krogue JD, Cheng KV, Hwang KM, et al. Automatic Hip Fracture Identification and Functional Subclassification with Deep Learning. *Radiology: Artificial Intelligence* 2020; **2**(2): e190023.
74. Kukar M, Kononenko I, Silvester T. Prognosing the femoral neck fracture recovery with machine learning. 1998.
75. Kumar B, Gill KS, Upadhyay D, Devliyal S. Revolutionizing Bone Injury Diagnosis with Advanced Deep Learning Approaches. 2024/8/29/: IEEE. p. 1–5.
76. Lam V, Parida A, Dance S, Tabaie S, Cleary K, Anwar SM. Analyzing pediatric forearm X-rays for fracture analysis using machine learning. *Int J CARS* 2025; **20**(9): 1845-50.
77. Langerhuizen DWG, Bulstra AEJ, Janssen SJ, et al. Is Deep Learning On Par with Human Observers for Detection of Radiographically Visible and Occult Fractures of the Scaphoid? *Clin Orthop Relat Res* 2020; **478**(11): 2653-9.
78. Langerhuizen DWG, Janssen SJ, Mallee WH, et al. What Are the Applications and Limitations of Artificial Intelligence for Fracture Detection and Classification in Orthopaedic Trauma Imaging? A Systematic Review. *Clin Orthop Relat Res* 2019; **477**(11): 2482-91.
79. Lee C, Jang J, Lee S, Kim YS, Jo HJ, Kim Y. Classification of femur fracture in pelvic X-ray images using meta-learned deep neural network. *Scientific Reports* 2020; **10**(1).
80. Lee KM, Lee SY, Han CS, Choi SM. Long bone fracture type classification for limited number of CT data with deep learning. 2020/3/30/: ACM. p. 1090-5.
81. Lee S, Kang WS, Seo S, et al. Model for Predicting In-Hospital Mortality of Physical Trauma Patients Using Artificial Intelligence Techniques: Nationwide Population-Based Study in Korea. *J Med Internet Res* 2022; **24**(12): e43757.
82. Lee SH, Han CS, Choi SM, Lee KM. Classification of Multiple Fractures Using Deep Learning. *JKIIS* 2019; **29**(4): 285 − 90.
83. Lee SH, Jeon J, Lee GJ, Park JY, Kim YJ, Kim KG. Automated Association for Osteosynthesis Foundation and Orthopedic Trauma Association classification of pelvic fractures on pelvic radiographs using deep learning. *Scientific Reports* 2024; **14**(1).
84. Lex JR, Di Michele J, Koucheki R, Pincus D, Whyne C, Ravi B. Artificial Intelligence for Hip Fracture Detection and Outcome Prediction. *JAMA Network Open* 2023; **6**(3): e233391.
85. Lim J, Chang S, Kim K, Park HJ, Kim E, Hong SW. Machine learning-based prediction of the necessity for the surgical treatment of distal radius fractures. *J Orthop Surg Res* 2025; **20**(1).
86. Lind A, Akbarian E, Olsson S, et al. Artificial intelligence for the classification of fractures around the knee in adults according to the 2018 AO/OTA classification system. *PLOS ONE* 2021; **16**(4): e0248809.
87. Lindsey R, Daluiski A, Chopra S, et al. Deep neural network improves fracture detection by clinicians. *Proceedings of the National Academy of Sciences* 2018; **115**(45): 11591-6.
88. Liu P-r, Zhang J-y, Xue M-d, et al. Artificial Intelligence to Diagnose Tibial Plateau Fractures: An Intelligent Assistant for Orthopedic Physicians. *CURR MED SCI* 2021; **41**(6): 1158-64.
89. Liu X-S, Nie R, Duan A-W, et al. YOLOX-SwinT algorithm improves the accuracy of AO/OTA classification of intertrochanteric fractures by orthopedic trauma surgeons. *Chinese Journal of Traumatology* 2025; **28**(1): 69–75.
90. Liu Y, Cheng L. Ultrasound Images Guided under Deep Learning in the Anesthesia Effect of the Regional Nerve Block on Scapular Fracture Surgery. *Journal of Healthcare Engineering* 2021; **2021**: 1–10.
91. Long Z, Tan S, Sun B, et al. PREDICTING IN-HOSPITAL MORTALITY IN CRITICAL ORTHOPEDIC TRAUMA PATIENTS WITH SEPSIS USING MACHINE LEARNING MODELS. *Shock* 2024; **63**(6): 815 − 25.
92. Lu E, Dubose J, Venkatesan M, et al. Using machine learning to predict outcomes of patients with blunt traumatic aortic injuries. *J Trauma Acute Care Surg* 2024; **97**(2): 258 − 65.
93. Machine Learning Consortium obotS, Investigators F. A Machine Learning Algorithm to Identify Patients with Tibial Shaft Fractures at Risk for Infection After Operative Treatment. Journal of Bone and Joint Surgery American volume: Ovid Technologies (Wolters Kluwer Health); 2020.
94. Magnéli M, Ling P, Gislén J, et al. Deep learning classification of shoulder fractures on plain radiographs of the humerus, scapula and clavicle. *PLOS ONE* 2023; **18**(8): e0289808.
95. Maurer LR, Bertsimas D, Bouardi HT, et al. Trauma outcome predictor: An artificial intelligence interactive smartphone tool to predict outcomes in trauma patients. *J Trauma Acute Care Surg* 2021; **91**(1): 93 − 9.
96. Meadi MN, El Mouméne Zerari A, Benbrahim H. Cervical spine fracture detection using deep learning algorithm. 2024/4/21/: IEEE. p. 1–8.
97. Merrill RK, Ferrandino RM, Hoffman R, Shaffer GW, Ndu A. Machine Learning Accurately Predicts Short-Term Outcomes Following Open Reduction and Internal Fixation of Ankle Fractures. *The Journal of Foot and Ankle Surgery* 2019; **58**(3): 410-6.
98. Millarch AS, Folke F, Rudolph SS, Kaafarani HM, Sillesen M. Prehospital triage of trauma patients: predicting major surgery using artificial intelligence as decision support. *British Journal of Surgery* 2025; **112**(4).
99. Misbah I, Sekar A, Muthu S, et al. Automatic distal radius fracture detection and classification using deep convolutional neural network with radiological images. *J Autonom Intell* 2024; **7**(5): 1632.
100. Murphy EA, Ehrhardt B, Gregson CL, et al. Machine learning outperforms clinical experts in classification of hip fractures. *Scientific Reports* 2022; **12**(1).
101. Naseem M. 513 A Deep Learning Computer Vision Injury Prediction tool (MALTA)-Pilot study. 2022/11//: BMJ Publishing Group Ltd. p. A78.1-A.
102. Nassour N, Akhbari B, Ranganathan N, et al. Using machine learning in the prediction of symptomatic venous thromboembolism following ankle fracture. *Foot and Ankle Surgery* 2024; **30**(2): 110-6.
103. Ngan KH, d’Avila Garcez A, Knapp KM, Appelboam A, Reyes-Aldasoro CC. A machine learning approach for Colles’ fracture treatment diagnosis. *bioRxiv* 2020.
104. Niggli C, Pape HC, Mica L. Validation of IBM-WATSON Health Trauma Pathway explorer, a visual based analytics tool to predict outcomes of poly-trauma patients. 2020/10/15/: Georg Thieme Verlag KG.
105. Niño-Sandoval TC, Landim FS, Vasconcelos BCE. Deep learning for orbital fracture detection and reconstruction: A systematic review on diagnostic accuracy and surgical planning. *Journal of Cranio-Maxillofacial Surgery* 2025; **53**(9): 1501-10.
106. Olczak J, Emilson F, Razavian A, Antonsson T, Stark A, Gordon M. Ankle fracture classification using deep learning: automating detailed. *Acta Orthopaedica* 2020; **92**(1): 102-8.
107. Olczak J, Fahlberg N, Maki A, et al. Artificial intelligence for analyzing orthopedic trauma radiographs. *Acta Orthopaedica* 2017; **88**(6): 581-6.
108. Olczak J, Prijs J, Ijpma F, et al. External validation of an artificial intelligence multi-label deep learning model capable of ankle fracture classification. *BMC Musculoskeletal Disorders* 2024; **25**(1).
109. Oosterhoff JHF, Gravesteijn BY, Karhade AV, et al. Feasibility of Machine Learning and Logistic Regression Algorithms to Predict Outcome in Orthopaedic Trauma Surgery. *Journal of Bone and Joint Surgery* 2021; **104**(6): 544 − 51.
110. Ouyang C-H, Chen C-C, Tee Y-S, et al. The Application of Design Thinking in Developing a Deep Learning Algorithm for Hip Fracture Detection. *Bioengineering* 2023; **10**(6): 735.
111. Ozkaya E, Topal FE, Bulut T, Gursoy M, Ozuysal M, Karakaya Z. Evaluation of an artificial intelligence system for diagnosing scaphoid fracture on direct radiography. *Eur J Trauma Emerg Surg* 2020; **48**(1): 585 − 92.
112. Paydar S, Parva E, Ghahramani Z, et al. Do clinical and paraclinical findings have the power to predict critical conditions of injured patients after traumatic injury resuscitation? Using data mining artificial intelligence. *Chinese Journal of Traumatology* 2021; **24**(1): 48–52.
113. Praharsha CV, Srikanth P. Classification and Monitoring of Injuries Around Knee Using Radiograph-Based Deep Learning Algorithm. Studies in Big Data: Springer Singapore; 2021: 127 − 46.
114. Pranata YD, Wang K-C, Wang J-C, et al. Deep learning and SURF for automated classification and detection of calcaneus fractures in CT images. *Computer Methods and Programs in Biomedicine* 2019; **171**: 27–37.
115. Rayan JC, Reddy N, Kan JH, Zhang W, Annapragada A. Binomial Classification of Pediatric Elbow Fractures Using a Deep Learning Multiview Approach Emulating Radiologist Decision Making. *Radiology: Artificial Intelligence* 2019; **1**(1): e180015.
116. Regnard N-E, Lanseur B, Ventre J, et al. Assessment of performances of a deep learning algorithm for the detection of limbs and pelvic fractures, dislocations, focal bone lesions, and elbow effusions on trauma X-rays. *European Journal of Radiology* 2022; **154**: 110447.
117. Rezapour M, Seymour RB, Sims SH, Karunakar MA, Habet N, Gurcan MN. Employing machine learning to enhance fracture recovery insights through gait analysis. *Journal Orthopaedic Research* 2024; **42**(8): 1748-61.
118. Rodrigo M, Athuraliya B. FracturePro: Automated Bone Fracture Identification and Classification Using an Ensemble Deep Learning Approach. 2025/2/19/: IEEE. p. 1–6.
119. Rosenberg GS, Cina A, Schirò G, et al. ARTIFICIAL INTELLIGENCE ACCURATELY DETECTS TRAUMATIC 1 THORACOLUMBAR FRACTURES ON SAGITTAL RADIOGRAPHS 2. 2021.
120. Rosenberg GS, Cina A, Schiró GR, et al. Artificial Intelligence Accurately Detects Traumatic Thoracolumbar Fractures on Sagittal Radiographs. *Medicina* 2022; **58**(8): 998.
121. S G, G. A S, Lakshmi Priya P, S K. A Novel Revolutionizing Prediction of Bone Fracture using Deep Learning Techniques. 2025/3/19/: IEEE. p. 840-5.
122. Selitsky S. How Bayesian artificial intelligence help fight uncertainty in trauma survival predictions: analysis of concepts. *Medical news of the North Caucasus* 2023; **18**(2).
123. Shinohara I, Inui A, Mifune Y, et al. Ultrasound With Artificial Intelligence Models Predicted Palmer 1B Triangular Fibrocartilage Complex Injuries. *Arthroscopy: The Journal of Arthroscopic & Related Surgery* 2022; **38**(8): 2417-24.
124. Singh S, Vats S, Bhan A, Khan N. Implementation and optimization of Deep learning models for Musculoskeletal image classification for detection of Osteoporosis. 2023/6/8/: IEEE. p. 1–6.
125. Staziaki PV, Wu D, Rayan JC, et al. Machine learning combining CT findings and clinical parameters improves prediction of length of stay and ICU admission in torso trauma. *Eur Radiol* 2021; **31**(7): 5434-41.
126. Sudha I, Ramesh PS, Durgadevi P, Sundari G, S S, Narang S. FractureAI Revolutionizing Bone Fracture Detection with Deep Learning CNNs for Precision Medical Imaging. 2025/3/4/: IEEE. p. 1179-84.
127. Sultana T, Parveen AV. Efficient Bone Fracture Detection And Classification Using Machine Learning Approaches. 2022.
128. Tanzi L, Vezzetti E, Moreno R, Aprato A, Audisio A, Massè A. Hierarchical fracture classification of proximal femur X-Ray images using a multistage Deep Learning approach. *European Journal of Radiology* 2020; **133**: 109373.
129. Teng Y, Pan D, Zhao W. Application of deep learning ultrasound imaging in monitoring bone healing after fracture surgery. *Journal of Radiation Research and Applied Sciences* 2023; **16**(1): 100493.
130. Tesfaw A, Teshager A, Mirolgn A, Ambachew E. Bone Fracture Classification Using Heterogeneous Ensemble Machine Learning and Deep Learning Algorithms. *SSRN Journal* 2024.
131. Thomas D. Artificial Intelligence Based Wrist Fracture Classification [Doctoral dissertation]: Louisiana State University Libraries; 2019.
132. Thongpeth T. Predicting Mortality of Orthopedic Trauma in Road Traffic Injury.(การทำานายโอกาสการเสียชีวิตในผู้บาดเจ็บทางกระดูกและข้อจากอุบัติเหตุทางการจราจร). 2018.
133. Tümen L, Medved F, Rachunek-Medved K, Han Y, Saul D. Deep Learning in Scaphoid Nonunion Treatment. *Journal of Clinical Medicine* 2025; **14**(6): 1850.
134. Udombuathong P, Srisawasdi R, Kesornsukhon W, Ratanasanya S. APPLICATION OF ARTIFICIAL INTELLIGENCE TO ASSIST HIP FRACTURE DIAGNOSIS USING PLAIN RADIOGRAPHS. *J Southeast Asian Med Res* 2022; **6**: e0111.
135. Urakawa T, Tanaka Y, Goto S, Matsuzawa H, Watanabe K, Endo N. Detecting intertrochanteric hip fractures with orthopedist-level accuracy using a deep convolutional neural network. *Skeletal Radiol* 2018; **48**(2): 239 − 44.
136. Vasker N, Haider SN, Hasan M, Uddin MS. Deep Learning-assisted Fracture Diagnosis: Real-time Femur Fracture Diagnosis and Categorization. 2023/8/25/: IEEE. p. 1–6.
137. Wang S. Study on the application of deep learning artificial intelligence techniques in the diagnosis of nasal bone fracture. *Int J Burn Trauma* 2024; **14**(6): 125 − 32.
138. White J, Wadhawan A, Min H, et al. DISTAL RADIUS FRACTURE CLASSIFICATION USING DEEP LEARNING ALGORITHMS. *Bone Joint J* 2023; **105-B**(SUPP_2): 102-.
139. Xie Y, Chen X, Yang H, et al. Integrating blockchain technology with artificial intelligence for the diagnosis of tibial plateau fractures. *Eur J Trauma Emerg Surg* 2025; **51**(1).
140. Xie Y, Wang X, Yang H, et al. Swarm learning network for privacy-preserving and collaborative deep learning assisted diagnosis of fracture: a multi-center diagnostic study. *Front Med* 2025; **12**.
141. Xing P, Zhang L, Wang T, Wang L, Xing W, Wang W. A deep learning algorithm that aids visualization of femoral neck fractures and improves physician training. *Injury* 2024; **55**(12): 111997.
142. Yang F, Ding B. Computer Aided Fracture Diagnosis Based on Integrated Learning. 2020/9/27/: IEEE. p. 523-7.
143. Yang S, Yin B, Cao W, Feng C, Fan G, He S. Diagnostic accuracy of deep learning in orthopaedic fractures: a systematic review and meta-analysis. *Clinical Radiology* 2020; **75**(9): 713.e17-.e28.
144. Yoon AP, Chung KC. Application of deep learning: detection of obsolete scaphoid fractures with artificial neural networks. *J Hand Surg Eur Vol* 2021; **46**(8): 914-6.
145. Zhang X, Yang Y, Shen Y-W, et al. Diagnostic accuracy and potential covariates of artificial intelligence for diagnosing orthopedic fractures: a systematic literature review and meta-analysis. *Eur Radiol* 2022; **32**(10): 7196 − 216.
146. Zheng Z, Ryu BY, Kim SE, et al. Deep learning for automated hip fracture detection and classification. *Bone Joint J* 2025; **107-B**(2): 213 − 20.
